# Factors Associated with the Accuracy of Sonographic Fetal Weight Estimation: A Study of 1262 Vietnamese Pregnancies

**DOI:** 10.3390/diagnostics16213457

**Published:** 2026-10-23

**Authors:** Ngoc Diem Doan Phan, Quang Chi Nguyen, Trang Thi Thu Nguyen, Xuan Thi Huong Nguyen, Nhung Huynh Ho, Quang Sy Nguyen, Thuy Thi Chau, Duong Huu Quach, Tuyen Kim Cat Vo

**Affiliations:** 1 Department of Obstetrics and Gynecology, Vinmec Central Park International Hospital, Vinmec International Hospital, Ho Chi Minh City 70000, Vietnam; v.ngocpdd@vinmec.com (N.D.D.P.); v.quangnc@vinmec.com (Q.C.N.); v.trangntt57@vinmec.com (T.T.T.N.); v.xuannth1@vinmec.com (X.T.H.N.); v.nhunghh7@vinmec.com (N.H.H.); v.quangns3@vinmec.com (Q.S.N.); v.thuyct12@vinmec.com (T.T.C.); v.duongqh1@vinmec.com (D.H.Q.); 2 College of Health Sciences, VinUniversity, Hanoi 10000, Vietnam; 3 School of Medicine, University of Medicine and Pharmacy at Ho Chi Minh City, Ho Chi Minh City 70000, Vietnam

**Keywords:** fetal weight estimation, Hadlock formula, Shinozuka formula, obstetric ultrasound, fetal biometry, macrosomia, Vietnam

## Abstract

**Background/Objectives:** Sonographic estimated fetal weight (EFW) guides many obstetric decisions. We examined maternal, fetal and sonographic factors associated with EFW error using the Hadlock II, Hadlock III and Shinozuka formulas. **Methods:** This is a retrospective cross-sectional study of 1262 singleton pregnancies at 30–41 weeks scanned within 3 days before delivery. Accuracy was assessed by mean percentage error (MPE), mean absolute percentage error (MAPE), successful estimation rate (SER; error ≤ 10%), Bland–Altman analysis with regression-based limits of agreement and Deming regression. Formulas were compared with paired tests. Each factor’s contribution was measured as the decrease in Nagelkerke R^2^ (ΔR^2^) in multivariable logistic regression; factor × formula interactions were tested with generalised estimating equations (GEE). **Results:** MAPE was 5.44%, 5.64% and 5.75% and SER 86.2%, 84.2% and 84.5% for Hadlock II, Hadlock III and Shinozuka, respectively; differences between formulas were small (ΔMAPE ≤ 0.31 percentage points). Against birth weight, all three overestimated small and underestimated large fetuses (Deming slopes 0.79–0.85). Birth weight was the strongest correlate of SER (decrease in Nagelkerke R^2^, 0.052–0.129) and, in the main analysis, the only factor whose effect differed between formulas (*p* < 0.001); other factors reduced Nagelkerke R^2^ by <0.02. Sensitivity was 42–66% for the 89 neonates < 2500 g and 18–38% for the 39 neonates ≥ 4000 g; positive predictive values were 78–82% and 88–100%, respectively, the latter based on 8–16 estimates (lower 95% confidence limits, 47–74%). **Conclusions:** EFW error was mainly associated with fetal size; this pattern, partly due to regression towards the mean, cannot be used as a bedside correction. Other characteristics showed little additional association with accuracy. The differences between the formulas were small, their clinical relevance is unclear, and none is recommended over the others. An EFW of 2500–2999 g does not exclude low birth weight, and an EFW of 3500–3999 g does not exclude a neonate of ≥4000 g.

## 1. Introduction

Prenatal estimation of fetal weight is one of the most important tasks in pregnancy management. Fetal weight directly influences decisions on the timing and mode of delivery and on the detection of macrosomia or fetal growth restriction, thereby helping to reduce obstetric complications and perinatal mortality. However, its performance is limited: recent meta-analyses report that third-trimester ultrasound identifies only half to three quarters of large-for-gestational-age or macrosomic fetuses [1,2]. In a multicentre cohort of 3992 pregnancies, EFW alone at 35–36 weeks of gestation identified only 47.9% of infants below the 10th centile [3].

Ultrasound is currently the standard tool for estimating fetal weight. The Hadlock II [4], Hadlock III [4] and Shinozuka [5] formulas are commonly used in clinical practice, are preinstalled on ultrasound machines and have demonstrated high accuracy in many studies [6,7,8,9]. However, the accuracy of these formulas is not fixed and may vary across clinical situations. Many studies have examined how maternal, fetal and sonographic factors affect the accuracy of fetal weight estimation, but their conclusions remain inconsistent and debated [10,11,12,13]. The reported effects of maternal size, diabetes, fetal sex, presentation and amniotic fluid differ between populations. Most studies also tested only one formula, so it is unclear whether a factor affects all formulas or only one. We therefore conducted this study to identify which characteristics are associated with the error of a sonographic fetal weight estimate and whether these associations differ between the three formulas.

## 2. Materials and Methods

### 2.1. Study Population and Data Collection

**Figure 1:**
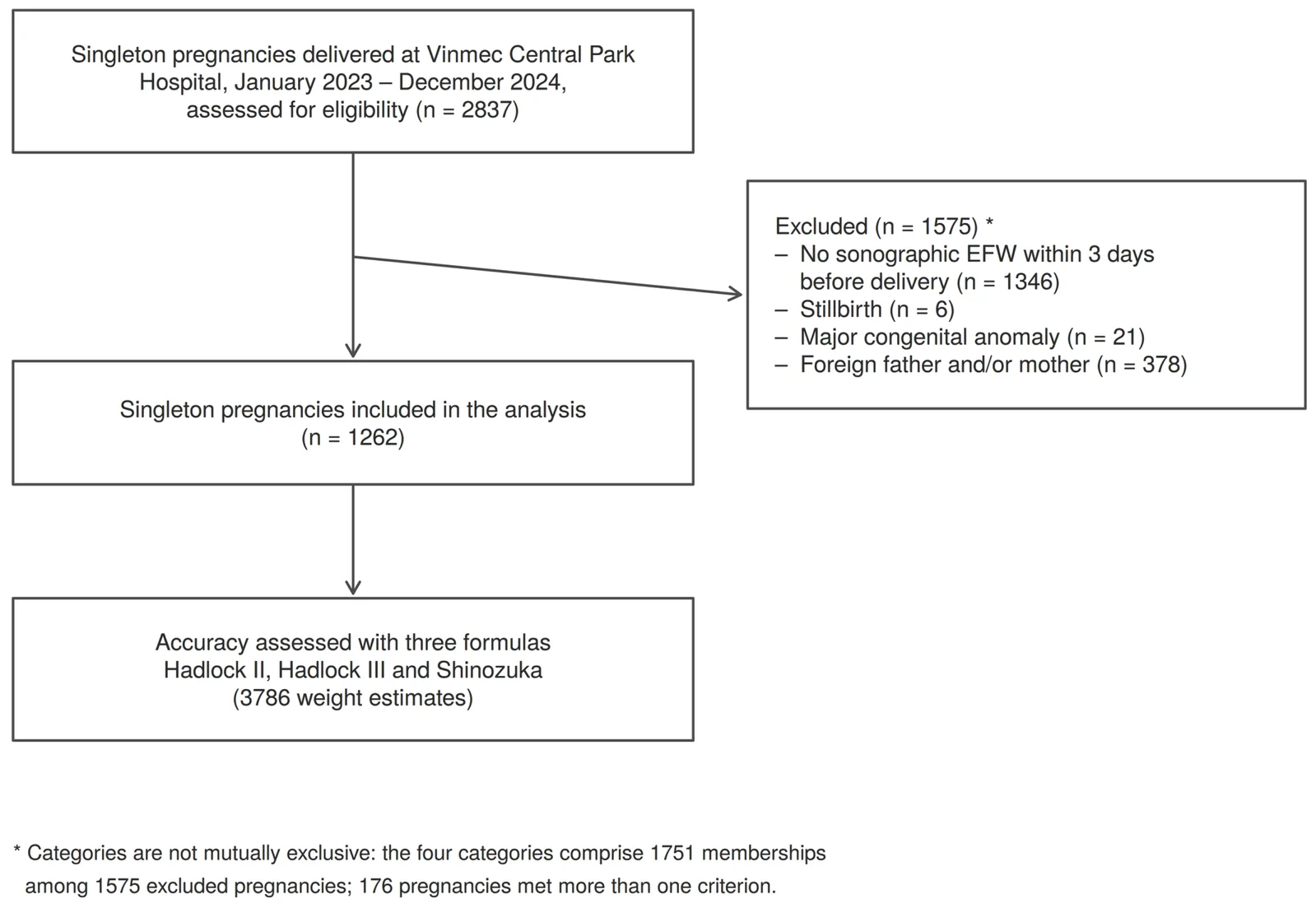
Flow diagram of the study population.

This retrospective cross-sectional study, reported in accordance with the STROBE statement, collected sonographic and obstetric data from all women with a singleton pregnancy at 30–41 weeks of gestation (dated by crown–rump length measured between 7 weeks and 13 weeks 6 days) who delivered at Vinmec Central Park Hospital between January 2023 and December 2024 and who had a sonographic EFW within 3 days before delivery. We excluded stillbirths, fetuses with major congenital anomalies and cases in which the father and/or the mother was a foreign national. We applied this criterion so that the cohort would represent one population (Vietnamese women). It does not imply that nationality is a biological determinant of fetal growth. The selection of participants is shown in Figure 1. The same cohort is also used in a companion manuscript that compares the accuracy of 14 fetal weight estimation formulas and their performance across birth-weight groups [14]. The present study asks a different question: which maternal, fetal and sonographic factors are associated with the error. The two reports overlap only in the accuracy of the three formulas in the whole cohort and by birth-weight group (Section 3.2), which is shown here as background for the factor analysis.

The study was approved by the Ethics Committee of the University of Medicine and Pharmacy at Ho Chi Minh City (Approval No. 3378/ĐHYD-HĐĐĐ) and was conducted in accordance with the Declaration of Helsinki. Because the study used existing medical records retrospectively, the requirement for written informed consent was waived by the committee.

Obstetric data were obtained from the electronic medical records and included maternal age, height and weight at delivery, diabetes in pregnancy (a single variable comprising gestational diabetes mellitus [GDM] in 242 women and type 2 diabetes diagnosed before pregnancy in 2), birth weight and fetal sex. GDM was diagnosed with a one-step 75 g oral glucose tolerance test at 24–28 weeks according to the International Association of Diabetes and Pregnancy Study Groups criteria (fasting plasma glucose ≥ 5.1 mmol/L, 1 h ≥10.0 mmol/L or 2 h ≥8.5 mmol/L) [15]. Newborns received essential care and were weighed on an electronic scale integrated into the resuscitation trolley no later than 90 min after birth; the weight was measured three times and averaged.

### 2.2. Ultrasound Measurements and Weight Estimation Formulas

Biometric measurements—biparietal diameter (BPD), abdominal circumference (AC), head circumference (HC) and femur length (FL)—were obtained from the ultrasound examination performed within 3 days before delivery; when a woman was scanned more than once in this period, the scan closest to delivery was used, and the EFW was calculated in a Microsoft Excel for Microsoft 365 spreadsheet (Microsoft Corporation, Redmond, WA, USA) using the Hadlock II [4], Hadlock III [4] and Shinozuka [5] formulas (Table 1). Measurements followed the International Society of Ultrasound in Obstetrics and Gynecology (ISUOG) guidelines [16], and third-trimester examinations were performed in accordance with the corresponding ISUOG practice guideline [17]. BPD was measured on a transverse view of the head at the level of the thalami and cavum septi pellucidi, without passing through the cerebellum, from the outer edge of the proximal skull to the inner edge of the distal skull (outer–inner). HC was measured in the same plane using the ellipse method, with calipers placed at the outer border of the skull. AC was measured on a transverse section of the abdomen at the junction of the umbilical vein and portal sinus, avoiding sections through the kidneys or chest, using the ellipse method with calipers at the outer body contour. FL was measured along the straightest possible long axis, from the proximal end to the distal condyle, excluding the distal femoral epiphysis and soft tissue.

We also recorded fetal presentation, the amniotic fluid index (AFI) and the single deepest pocket (SDP). Polyhydramnios was defined as an AFI ≥ 24 cm and/or an SDP ≥ 8 cm and oligohydramnios as an AFI ≤ 5 cm and/or an SDP ≤ 2 cm. All scans were performed by two sonographers with more than 20 years of experience, using Voluson E8 and E10 machines (GE Healthcare, Chicago, IL, USA).

### 2.3. Statistical Analysis

The accuracy of each formula was assessed with several metrics. The percentage error (PE) was calculated as PE = (EFW − BW)/BW × 100; the mean percentage error (MPE) reflects whether a formula tends to over- or underestimate fetal weight. Because positive and negative values cancel out when averaged, we also used the absolute percentage error (APE = |PE| = |EFW − BW|/BW × 100) and the mean absolute percentage error (MAPE). The successful estimation rate (SER) was defined as the proportion of cases with an APE ≤ 10%; for the overall performance of each formula, we also report the proportions of cases with an APE ≤ 5% and ≤15%. Agreement between the estimated and the actual weight was displayed with Bland–Altman plots of the difference (EFW − BW) against birth weight, with the mean difference and fixed 95% limits of agreement (mean ± 1.96 SD). Because birth weight is a reference value obtained with an electronic scale rather than a second estimate of the same quantity, the difference was plotted against birth weight itself rather than against the mean of the two values [18]. As the difference varied systematically with fetal size, we additionally computed regression-based limits of agreement [19]: the difference was regressed on birth weight, the absolute residuals of that regression were regressed on birth weight and the limits were obtained as the fitted difference ± 2.46 times the fitted absolute residual. Confidence intervals were calculated for the mean bias (mean ± t_0.975;_ *_n_*_−1_ × SD/√*n*) and for each limit of agreement (limit ± t_0.975;_ *_n_*_−1_ × SD × √(3/*n*)). The slope of the difference on birth weight is reported with its 95% confidence interval and *p*-value; the slope of the absolute residuals on birth weight, with its 95% confidence interval, was used to describe whether the scatter in grams changed with fetal size. The regression-based limits are reported in grams and as a percentage of birth weight at 2500 g, at the mean birth weight and at 4000 g. As a complementary method-comparison analysis that does not assume birth weight to be measured without error, Deming regression of the EFW on birth weight was fitted with an error-variance ratio λ = 1, with 95% confidence intervals obtained from 2000 bootstrap resamples. Because the error of the scale is much smaller than that of ultrasound, the true ratio is at least 1; λ = 1 therefore gives the slope closest to unity within the plausible range and is a conservative test of proportional bias. Because the intercept at a birth weight of 0 g is an extrapolation far below the observed data, the Deming line was evaluated at the mean birth weight instead. Differences in slope between formulas were tested with a paired bootstrap: in each of 2000 resamples of women, the Deming and Bland–Altman slopes of all three formulas were estimated in the same resample, and the 95% confidence interval of each pairwise difference was taken from the 2.5th and 97.5th percentiles.

Because all three estimates were obtained in the same pregnancies, the formulas were compared by paired testing rather than descriptively. The absolute percentage errors of the three formulas were compared with the Friedman test and, pairwise, with the Wilcoxon signed-rank test, with 95% confidence intervals for the difference in MAPE obtained from 10,000 bootstrap resamples; the successful estimation rates were compared with Cochran’s Q test and, pairwise, with the McNemar test, with 95% confidence intervals for the paired difference in proportions. *p*-values for the three pairwise comparisons were adjusted by the Holm method.

Because only the EFW, not the birth weight, is known in clinical practice, and because examining the error against the true weight produces an apparent proportional bias through regression towards the mean even for an estimate that is correct on average for each value of the EFW, accuracy was also described by each formula’s own EFW groups, together with the slope of the difference (EFW − BW) on EFW, and the sensitivity and positive predictive value of an EFW < 2500 g and ≥4000 g for identifying neonates with the corresponding birth weight, with exact (Clopper–Pearson) 95% confidence intervals.

Nonparametric tests were used to compare the distribution of percentage errors between groups: the Mann–Whitney U test for two groups and the Kruskal–Wallis test for more than two groups. For descriptive comparability with previous reports, the MPE and MAPE are presented as mean ± standard deviation (SD). The chi-square (χ^2^) or Fisher exact test was used to compare the SER between groups. Multivariable binary logistic regression with a backward stepwise likelihood-ratio method (removal at *p* > 0.10; last category of each factor as the reference) was used to assess the independent association of each factor with the SER. In the multivariable models, strata containing fewer than 25 pregnancies were combined with the adjacent stratum to avoid unstable estimates: gestational age was entered as a single category below 37 weeks, maternal height as <160, 160–169.9 and ≥170 cm, and maternal weight at delivery as <60, 60–69.9, 70–79.9 and ≥80 kg. The descriptive and univariate results are reported for the original, finer strata. To quantify the individual contribution of each factor, the Nagelkerke R^2^ of the full model (enter method) was compared with that of models omitting each factor in turn (ΔR^2^). Nagelkerke R^2^ is a pseudo-R^2^ and not a proportion of explained variance; ΔR^2^ was therefore used only to compare the contribution of the factors. Because each formula was analysed in a separate model, comparisons of ΔR^2^ between formulas are approximate. To test whether the effect of each factor differed between the three formulas, all three formulas were entered into a single model rather than compared as three separate models. Each woman contributed three records, one per formula, and a generalised estimating equations model (GEE; binomial family, logit link, exchangeable working correlation, woman as the clustering unit, robust standard errors) was used to account for the three records belonging to the same woman. For each factor, a factor × formula interaction term was added to the nine-factor model and assessed with a Wald test; *p*-values for the nine tests were adjusted by the Holm method. In the GEE model, the factors were coded in the same merged strata as in the logistic models. Because the gestational-age strata were merged most heavily, the gestational age × formula interaction was also tested with gestational age in four categories (<34, 34–36 + 6, 37–39 + 6 and ≥40 weeks) as a sensitivity analysis. For significant interactions, the odds ratio for each category was estimated separately for each formula (for birth weight, with 3000–3499 g as the reference).

Two sensitivity analyses were performed. First, because birth weight is the denominator of the percentage error, it is part of both the outcome and the predictor set. The same nine-factor model was therefore refitted with two continuous outcomes: the absolute percentage error and the absolute error in grams, which does not have birth weight in the denominator. Second, because the scan-to-delivery interval confounds estimation error with true fetal growth, accuracy was recalculated for scans performed on the day of delivery and within one and two days before delivery and after multiplying each estimate by (1 + 0.008 × interval in days). The interval was calculated as the difference between the calendar dates of the scan and of delivery, because the times of the scans were not available; an interval of 0 days therefore means a scan on the day of delivery, and the true interval may differ from the recorded value by up to about one day, so the growth correction is approximate. The coefficient of 0.008 corresponds to approximately 25 g per day at the cohort’s mean weight and was derived from a mean gain of 176.5 g per week in 12,425 term singleton pregnancies, third-trimester fetal growth being nearly linear [20]. The analysis was repeated with coefficients of 0.005 and 0.010 to check the robustness of the conclusions.

A *p*-value < 0.05 was considered statistically significant. Analyses were performed with SPSS version 23.0 (IBM Corp., Armonk, NY, USA) and R version 4.3.3 (R Foundation for Statistical Computing, Vienna, Austria) with the geepack package, version 1.3.10.

Use of generative artificial intelligence. A generative artificial intelligence assistant (Claude, Anthropic, San Francisco, CA, USA; Claude Opus 5, accessed August and September 2026) was used during the preparation of this manuscript. Its use was limited to drafting and language editing. It was not used to generate, analyse or interpret data. The authors conceived the study, performed the analyses, verified every AI-assisted sentence against the primary data and the cited sources and take full responsibility for the content of this article.

## 3. Results

### 3.1. Characteristics of the Study Population

The study included 1262 pregnancies, whose characteristics are shown in Table 2. The mean maternal age was 32.7 ± 4.2 years, the mean height 158.8 ± 5.2 cm and the mean weight at delivery 67.0 ± 8.6 kg. Diabetes in pregnancy was present in 244 women (19.3%): 242 with GDM and two with type 2 diabetes diagnosed before pregnancy. Most pregnancies were at term (37–41 weeks; 96.3%), and 45.4% of neonates weighed 3000–3499 g. All cases had a scan-to-delivery interval ≤ 3 days, with 46.0% of women scanned 1 day before delivery. Abnormal presentation, polyhydramnios and oligohydramnios were uncommon (4.9%, 3.4% and 2.3%, respectively). Data were complete for all variables included in the analyses.

### 3.2. Accuracy of the Estimates and Their Dependence on Fetal Size

The accuracy of the three formulas across the whole cohort is shown in Table 3. All three were reasonably accurate: the MAPE was below 6% (Hadlock II, 5.44 ± 4.49%; Hadlock III, 5.64 ± 4.48%; Shinozuka, 5.75 ± 4.79%), the successful estimation rate lay between 84% and 87% (86.2%, 84.2% and 84.5%, respectively) and the absolute percentage error was within 15% in more than 95% of cases. The formulas differed mainly in the direction of bias: Hadlock III systematically underestimated fetal weight (MPE, −1.47 ± 7.05%), and Shinozuka systematically overestimated it (+1.83 ± 7.26%), whereas Hadlock II was close to unbiased (−0.37 ± 7.05%).

Because all three estimates were obtained in the same pregnancies, the formulas were also compared by paired testing (Table 4). Every pairwise difference was smaller than 0.4 percentage points of MAPE and 2.1 percentage points of SER. Hadlock II differed significantly from Hadlock III in both measures and from Shinozuka in MAPE; these differences were detectable because the design was paired and the sample large. They were small, but because no threshold for clinical relevance or equivalence margin was set in advance, their clinical relevance is unclear. We therefore did not rank the formulas.

**Figure 2:**
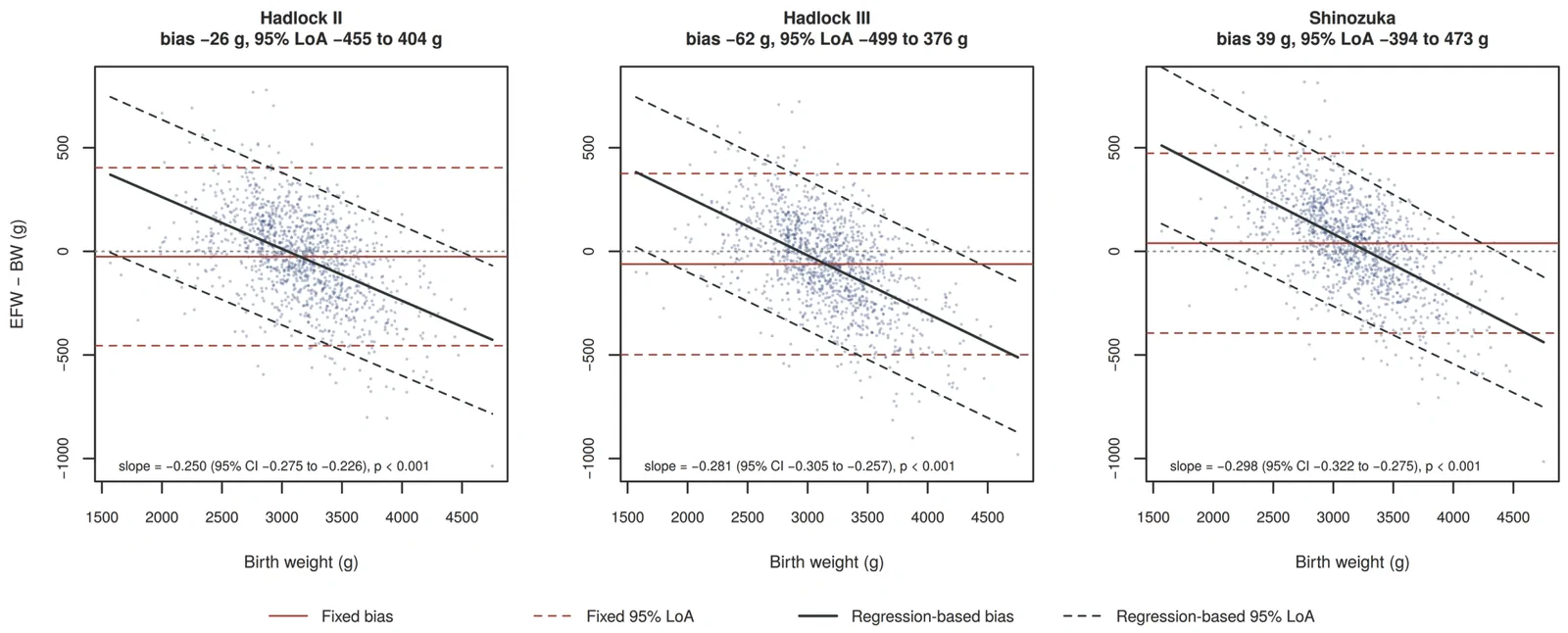
Bland–Altman plots of the difference between the estimated and the actual fetal weight (EFW − BW) against birth weight for the Hadlock II, Hadlock III and Shinozuka formulas (*n* = 1262). Red lines show the fixed mean difference (solid) and the fixed 95% limits of agreement (dashed, mean ± 1.96 SD). Black lines show the regression-based bias (solid) and the regression-based 95% limits of agreement (dashed). The slope of the difference on birth weight, with its 95% confidence interval, is given in each panel; all three slopes differ from zero at *p* < 0.001. Plots were produced in R. BW, birth weight; EFW, estimated fetal weight.

Figure 2 shows the Bland–Altman analysis, with the error expressed in grams. The mean difference between the estimated and the actual weight was −25.6 g for Hadlock II, −61.5 g for Hadlock III and +39.4 g for Shinozuka, but the 95% limits of agreement were wide and similar for the three formulas (Hadlock II, −455 to +404 g; Hadlock III, −499 to +376 g; Shinozuka, −394 to +473 g), spanning approximately 860–875 g in each case. In every formula, the difference declined steadily as birth weight increased (slope, −0.250 for Hadlock II, −0.281 for Hadlock III and −0.298 for Shinozuka; r = −0.49, −0.54 and −0.58, respectively), so that at the extremes of the observed weight range (1570 and 4752 g), the fitted difference was +370 to +510 g and −430 to −510 g, respectively. All three slopes differed significantly from zero (Hadlock II, −0.250; 95% CI, −0.275 to −0.226; Hadlock III, −0.281; −0.305 to −0.257; Shinozuka, −0.298; −0.322 to −0.275; all *p* < 0.001). The scatter of the differences, by contrast, did not widen with birth weight: regression of the absolute residuals on birth weight gave slopes of −0.0023 (95% CI, −0.0176 to +0.0130; *p* = 0.77), +0.0001 (−0.0148 to +0.0151; *p* = 0.99) and −0.0081 (−0.0226 to +0.0064; *p* = 0.27), so there was a proportional bias against birth weight but no evidence that the scatter of the differences in grams changed with birth weight; the confidence intervals exclude a change of more than about 35 g in the mean absolute residual between 2500 and 4000 g. Confidence intervals for the bias and for both limits of agreement are given in Table 5.

Because the difference varied with fetal size, regression-based limits of agreement were also computed (Figure 2, black lines). Between 2500 and 4000 g, their width in grams changed little (approximately 725–740 g for Hadlock II, 725 g for Hadlock III and 660–720 g for Shinozuka), but their centre shifted steeply with birth weight (Table 5). At a birth weight of 2500 g, the expected difference was +137 g (95% limits, −233 to +508 g) for Hadlock II, +121 g (−241 to +483 g) for Hadlock III and +233 g (−126 to +593 g) for Shinozuka; at 4000 g, the corresponding values were −238 g (−600 to +124 g), −300 g (−663 to +62 g) and −214 g (−543 to +115 g). Because the scatter in grams was similar, the same error in grams is a larger error relative to a small baby: the half-width of the regression-based limits was 14.4–14.8% of birth weight at 2500 g but 8.2–9.1% at 4000 g (Table 5).

Deming regression led to the same conclusion (Table 5). For all three formulas, the slope was significantly below unity (Hadlock II, 0.853; 95% CI, 0.821–0.882; Hadlock III, 0.817; 0.788–0.848; Shinozuka, 0.792; 0.761–0.823), confirming that, against birth weight, the estimates are compressed towards the middle of the weight range. The intercepts at 0 g (+439 g, +514 g and +696 g) are extrapolations far below the observed weights and were not interpreted; at the mean birth weight (3150 g), the Deming line gave differences of −25.6 g, −61.5 g and +39.4 g, equal to the mean bias. Although the confidence intervals of the slopes overlapped, the paired bootstrap showed that the slopes differed between formulas (Table 5): the Shinozuka slope was lower than the Hadlock II slope by 0.061 (95% CI, 0.056–0.066) and lower than the Hadlock III slope by 0.026 (0.016–0.036), and the Hadlock III slope was lower than the Hadlock II slope by 0.035 (0.027–0.043); the Bland–Altman slopes gave the same order. These differences are small: between 2500 and 4000 g, a difference in slope of 0.061 corresponds to about 90 g. The ordinary least-squares slopes, which correspond to a very large λ, were lower still (0.750, 0.719 and 0.702).

### 3.3. Accuracy by Estimated Fetal Weight

When cases were grouped by each formula’s own estimated weight rather than by birth weight, the size dependence of the error almost disappeared for the two Hadlock formulas (Table 6): the slope of the difference on the estimated weight was +0.013 (*p* = 0.418) for Hadlock II and −0.016 (*p* = 0.372) for Hadlock III, compared with −0.250 and −0.281 against birth weight. Shinozuka retained a small but significant slope (−0.049; *p* = 0.006). Grouping by birth weight and grouping by estimated weight answer different questions: the first shows how a formula performs in babies of a given true size, the second what an estimate means for the clinician, who knows only the EFW. Part of the size dependence against birth weight reflects regression towards the mean, which is expected when the error is examined against the true value rather than against the estimate (Section 4.2). The estimates were, however, compressed towards the middle of the weight range: only 7–15 of the 39 neonates weighing ≥ 4000 g were estimated at ≥4000 g (sensitivity, 17.9–38.5%), most of the remainder being estimated at 3500–3999 g (23–30 neonates), and only 37–59 of the 89 neonates weighing < 2500 g were estimated below 2500 g (sensitivity, 41.6–66.3%). Conversely, once the estimate crossed a threshold, the prediction was usually correct (Table 6 (B)): the positive predictive value (PPV) was 77.6–82.2% at 2500 g (45–76 estimates per formula) and 87.5–100% at 4000 g, but the latter rested on only 8–16 estimates and its lower 95% confidence limits were 47.3–73.5%.

### 3.4. Univariate Analysis

Factors affecting the accuracy of the formulas were analysed using the MPE, MAPE and SER (Table 7). Birth weight was the most consistent and pronounced factor, with statistically significant differences in the MPE, MAPE and SER for all three formulas. The SER was highest at 3000–3499 g (Hadlock II, 92.0%; Hadlock III, 90.6%; Shinozuka, 92.7%) and lowest in the two extreme groups (for example, 56.4% for Hadlock III at ≥4000 g and 60.7% for Shinozuka below 2500 g). All three formulas tended to overestimate weight in small fetuses (positive MPE) and underestimate it in large fetuses (negative MPE).

Maternal height affected the MPE of all three formulas and the MAPE of Hadlock III but not the SER; the ≥170 cm group had the largest negative MPE (Hadlock III, −6.19%) and the highest MAPE (7.14%), while Shinozuka was less affected in this group (SER, 86.5%). Maternal weight affected the MPE of all three formulas but not the MAPE or SER, with the MPE tending to decrease (from positive to negative) as maternal weight increased. Diabetes in pregnancy affected only the SER of Hadlock III (88.5% versus 83.1% in women without diabetes; *p* < 0.05). Gestational age affected the MPE of Hadlock II and both the MPE and MAPE of Hadlock III but not the SER of any formula and was not significant for Shinozuka. Fetal sex affected the MPE of all three formulas but not the MAPE or SER. Presentation affected only the MPE of Hadlock II and Shinozuka.

Amniotic fluid volume affected the SER of all three formulas, the MAPE of Hadlock III and the MPE of Shinozuka; the oligohydramnios group had the lowest SER (Hadlock II and III, 58.6%; Shinozuka, 62.1%) and the highest MAPE. The scan-to-delivery interval affected the MPE of all three formulas, the MAPE of Hadlock II and Hadlock III and the SER of Hadlock III; scans performed 3 days before delivery had the lowest SER with Hadlock III (75.3%), clearly lower than scans within 0–2 days (84.1–86.3%).

### 3.5. Multivariable Analysis

Results of the multivariable logistic regression (backward stepwise likelihood ratio) are shown in Table 8. In the final models, birth weight and amniotic fluid volume were independently and significantly associated with the SER of all three formulas. For birth weight, with ≥4000 g as the reference, the odds of a successful estimate in the 3000–3499 g group were 5.4 (Hadlock II), 8.2 (Hadlock III) and 5.1 (Shinozuka) times higher. For amniotic fluid, with oligohydramnios as the reference, the normal group had a significantly higher SER for all three formulas (odds ratio [OR], 3.94 and 4.22 for Hadlock II and Hadlock III, respectively; OR, 2.79 for Shinozuka).

The scan-to-delivery interval and diabetes in pregnancy were independently and statistically significantly associated with the SER only for Hadlock III. Scans within 0–2 days before delivery had 2.1–2.3 times higher odds of a successful estimate than scans 3 days before delivery (OR, 2.06–2.29). For Shinozuka, the interval was retained in the backward stepwise model (*p* = 0.084) but was not significant at the 0.05 level; only the 2-day group differed from the 3-day group (OR, 1.77; 95% CI, 1.00–3.13). Diabetes in pregnancy was independently associated with the SER of Hadlock III (OR, 0.62; 95% confidence interval [CI], 0.40–0.97; *p* = 0.036), women without diabetes having lower odds of a successful estimate than those with diabetes. Gestational age was independently associated with the SER only for Shinozuka, in which both groups below 40 weeks had higher odds of a successful estimate than the ≥40-week group after adjusting for weight (OR, 2.15 at 37–39 + 6 weeks and 5.70 before 37 weeks).

The final Nagelkerke R^2^ values were 0.074 (Hadlock II), 0.107 (Hadlock III) and 0.153 (Shinozuka). Birth weight was the largest contributor in all three models (decrease in Nagelkerke R^2^, 0.052 for Hadlock II, 0.064 for Hadlock III and 0.129 for Shinozuka); because each formula was analysed in a separate model, the larger value for Shinozuka indicates only approximately that its error was more strongly associated with birth weight. Removing any other factor reduced Nagelkerke R^2^ by 0.006 to 0.017.

The GEE model comprised 3786 estimates in 1262 women (Table 9). After adjustment for the nine factors, the odds of a successful estimate with Hadlock III and Shinozuka were 0.84 (95% CI, 0.75–0.94; *p* = 0.003) and 0.87 (0.76–0.99; *p* = 0.037) times those with Hadlock II, respectively, in the same direction as the paired comparisons in Table 4. In the main analysis, of the nine factor-by-formula interactions, only that with birth weight remained significant after Holm adjustment (χ^2^ = 68.6, df = 8, *p* < 0.001). Before adjustment, *p* was below 0.05 for maternal height (0.014), fetal sex (0.038) and gestational age (0.040), 0.064 for the scan-to-delivery interval and above 0.05 for the other four factors; after Holm adjustment, none was significant. These results do not show that the factors act equally on all formulas; they show only that no difference was detected, and several subgroups were small. In a sensitivity analysis with gestational age in four categories, the gestational age × formula interaction was also significant after Holm adjustment (Table 9, footnote); this result depended on how gestational age was grouped and rested on nine pregnancies delivered before 34 weeks. The difference by fetal size lay at the two ends of the weight distribution: relative to the 3000–3499 g group, Shinozuka lost the most accuracy in fetuses below 3000 g (OR, 0.12 at <2500 g and 0.24 at 2500–2999 g), Hadlock III lost the most in fetuses of 3500 g or more (OR, 0.29 and 0.12), and Hadlock II was not the most affected formula in any weight group.

Because the percentage error is defined relative to birth weight, birth weight enters both the outcome and the predictor set. We therefore refitted the same nine-factor model with the absolute percentage error and with the absolute error in grams—a scale that does not contain birth weight in a denominator—as continuous outcomes (Table 10). Birth weight remained the largest single contributor under every outcome definition (decrease in R^2^, 0.038–0.150 for the absolute percentage error and 0.062–0.118 for the absolute error in grams), and no other factor exceeded 0.02. The association with birth weight was therefore still present when the error was expressed in grams, so it is not only a result of dividing by birth weight. This does not rule out other effects of how the analysis is constructed: an error examined against the true weight can depend partly on that weight through regression towards the mean (Section 3.3), and it does not show the mechanism or cause and effect.

Because the interval between the scan and delivery allows real fetal growth, part of the difference between the estimate and the birth weight after two or three days is physiological gain rather than error. Table 11 shows that this contributes little here. Restricting the cohort to scans performed on the day of delivery did not reduce the error (MAPE of Hadlock II, 5.78% versus 5.44% overall). We used a correction of 0.8% per day, based on the near-linear third-trimester fetal growth reported in 12,425 term singleton pregnancies [20]. The systematic bias shifted as expected (MPE of Hadlock II from −0.37% to +0.64%) while the magnitude of the error remained almost unchanged (MAPE from 5.44% to 5.47%). Because the expected daily growth cannot be known precisely in an individual pregnancy, the correction was repeated with factors of 0.5% and 1.0% per day. With these factors, the MAPE of Hadlock II changed only from 5.44% to 5.51% and the SER from 86.7% to 86.1%, while the MPE shifted from +0.26% to +0.90%. Thus, for Hadlock II, correcting for growth changed the bias but not the size of the error, whichever factor was used; for Shinozuka, which already overestimated, it increased the error (Table 11).

## 4. Discussion

### 4.1. Principal Findings

The differences in accuracy between the three formulas were small (MAPE, 5.44–5.75%; SER, 84.2–86.2%), although some were statistically significant (Table 4), and the formulas differed mainly in the direction of their bias: Hadlock III tended to underestimate, and Shinozuka tended to overestimate. Because no threshold for a clinically relevant difference was set in advance, the clinical relevance of these differences is unclear, and none of the three can be recommended over the others on grounds of accuracy. Their SERs are at the upper end of recent large series—76.0% in 31,521 Swedish pregnancies [21] and 82.4% for Hadlock III in 268 Vietnamese term pregnancies [22]. The fixed 95% limits of agreement spanned about 860–875 g, so an estimate of 3200 g was compatible with a true weight of roughly 2800–3650 g. The regression-based limits kept a similar width in grams but shifted with fetal size (Figure 2), so in relative terms, they were wider for small babies (about ±14.5% of birth weight at 2500 g versus ±9% at 4000 g; Table 5).

Fetal size was the factor most strongly associated with error. Removing birth weight reduced the Nagelkerke R^2^ by 0.052–0.129, removing any other factor by at most 0.017, and the final models had Nagelkerke R^2^ values of only 0.074–0.153. Barel et al. [11] reported similarly low values (R^2^, 4.2–9.7%). Because Nagelkerke R^2^ is a pseudo-R^2^, these values cannot be read as the proportion of the variation explained; they show only that the measured factors were weakly associated with a successful estimate. Caliper placement, plane selection, image quality and sonographer skill may also contribute to the error, but they were not recorded in this study, so this remains a hypothesis. In the main GEE analysis, fetal size was the only factor whose interaction with formula was significant after adjustment for multiple testing (Table 9); in a sensitivity analysis, the interaction with gestational age was also significant, but this depended on how gestational age was grouped and rested on nine pregnancies before 34 weeks (Section 4.3). For the other factors, these tests did not detect a difference between formulas, which is not the same as showing that the factors act equally on all formulas. In these models, maternal characteristics showed little additional association with estimation accuracy after accounting for the other variables. Whether they would improve the formulas would need a separate analysis, with model development and validation, using only information available before birth.

One caution applies to the comparison of formulas: Hadlock II and Shinozuka use the biparietal diameter, whereas Hadlock III uses the head circumference. Near term, when the head is engaged and moulded, the plane needed for the head circumference is harder to obtain than that for the biparietal diameter, and the small disadvantage of Hadlock III may reflect acquisition rather than the formula itself. Automated biometry measures the biparietal diameter, head circumference and occipitofrontal diameter reproducibly (intraclass correlation coefficients above 0.75), whereas more complex intracranial structures, such as the cisterna magna and the lateral ventricular width, are less reproducible [23].

### 4.2. Birth Weight

As in many previous studies, accuracy was lowest in the smallest and largest fetuses [6,10,11,13,22,24,25,26]; in Hammami et al. [6], for example, the SER of Hadlock III fell from 79.9% overall to 72.8% below 2500 g. All three formulas overestimated small fetuses and underestimated large ones when the error was examined against birth weight, as others have reported [10,11,25]. Two mechanisms contribute. First, equations fitted by regression, such as the Hadlock formulas, shrink their estimates towards the mean of the derivation sample. Second, an estimate that is correct on average for each value of the EFW—as a regression equation is designed to be—still shows this pattern when its error is plotted against the true weight, because the neonates at the extremes of birth weight are, on average, those whose estimates erred towards the middle (regression towards the mean). Consistent with this, when cases were grouped by estimated weight, the only information available before birth, the two Hadlock formulas showed almost no size-dependent bias (Table 6). The proportional bias against birth weight therefore cannot be used as a bedside correction: for the Hadlock formulas, a correction based on the slope against birth weight would add bias to the EFW rather than remove it.

Shinozuka is a volume model rather than a regression equation: it treats the fetus as a head proportional to BPD^3^ and a trunk proportional to AC^2^·FL [5]. It should therefore be less affected by the first mechanism. It was not less size-dependent: its Deming slope was slightly lower than those of the Hadlock formulas (Table 5), and, unlike the Hadlock formulas, it remained size-dependent when cases were grouped by estimated weight (Table 6), so its estimates were compressed more than regression towards the mean alone would explain. One possible explanation is that the cube and square terms amplify measurement error—a 5% error in BPD becomes about 16% in BPD^3^—but we did not measure this directly. The differences between the formulas were small, and with all three formulas, small fetuses may be smaller than estimated, and large fetuses may be larger.

Clinically, this compression means that small and large fetuses are identified asymmetrically: once the EFW crosses a threshold, it is usually correct, but many neonates beyond the threshold are missed (Table 6 (B)). Our sensitivities were lower than in other reports—third-trimester ultrasound detects about half to three-quarters of large fetuses [1,2,27], and an EFW below 2500 g had a sensitivity of 69.7% and a positive predictive value of 91.4% in 269 pregnancies [28]—partly because most of our large neonates only just exceeded the threshold (29 of the 39 weighed less than 4250 g), so a small underestimation was enough to miss them. The high positive predictive value at 4000 g rests on only 8–16 estimates (lower 95% confidence limits, 47–74%) and needs confirmation in larger cohorts. In practice, an EFW below 2500 g usually indicates a low-birth-weight neonate, and an EFW of ≥4000 g probably indicates a large one; an EFW on the other side of the threshold does not exclude either condition. With an estimate of 3500–3999 g, particularly with gestational diabetes or another risk factor for macrosomia, a neonate of ≥4000 g should still be considered; with an estimate of 2500–2999 g, low birth weight cannot be excluded.

For clinicians, the differences between these three formulas were small: their overall accuracy and limits of agreement were similar, and their small differences in bias and size dependence point in different directions. Their clinical relevance is unclear, and we do not recommend one formula over the others; what matters more is to know the direction of the bias of the formula in use and to interpret estimates near the 2500 g and 4000 g thresholds with caution.

### 4.3. Other Factors

*Maternal height and weight.* Maternal height and weight at delivery were associated with the direction of the error (MPE) but not with the SER, in line with most recent studies [13,29,30]; in a Swedish population-based study, Granfors et al. [13] found no effect of maternal body mass index. Some studies did find an effect [11,31]. In Barel et al. [11], the Sabbagha [32] and Combs [33] formulas tended to underestimate as maternal weight and height increased, possibly because heavier women had more large fetuses, and Ghosal et al. [31] found that body mass index affected the detection of small- but not large-for-gestational-age fetuses.

*Gestational age.* Gestational age was not independently associated with the SER of Hadlock II or Hadlock III, consistent with other studies after adjustment for fetal weight [10,12,34], although Granfors et al. [13] found more unsuccessful estimates in the most preterm group (adjusted risk ratio, 1.30; 95% CI, 1.13–1.50). For Shinozuka, gestational age was retained as an independent factor in the multivariable model (ΔR^2^, 0.015). This shows an association within Shinozuka; it does not show that gestational age acts differently on Shinozuka than on the Hadlock formulas. The GEE interaction test was not significant after Holm adjustment (*p* = 0.040; adjusted *p* = 0.266), but it became significant when the nine pregnancies delivered before 34 weeks were analysed as a separate category (Table 9, footnote); in these nine pregnancies, Shinozuka was successful in eight and each Hadlock formula in six (Table 7). The evidence for a formula-specific effect of gestational age is therefore inconclusive and needs confirmation in a cohort with more preterm deliveries.

*Fetal sex.* Fetal sex affected only the direction of the error (MPE) and was not associated with the SER. This agrees with several reports [12,35], whereas others found differences in either direction [10,11,13,36], possibly because male fetuses are heavier and were over-represented in the populations used to derive the regression formulas [36].

*Fetal presentation.* Presentation also affected only the direction of the error and was not associated with the SER, in line with several studies [10,12,37,38]; others found better accuracy in cephalic than in non-cephalic presentations [13,39,40], perhaps because a breech or transverse fetus offers a less favourable view and is scanned less often.

*Diabetes in pregnancy.* The prevalence of GDM was fairly high (242 women, 19.2%), possibly because the data came from a private tertiary hospital, but it was comparable to that reported from other Vietnamese hospitals (18.0–22.8%) [41,42,43]. Diabetes in pregnancy did not increase the error: it was associated with a higher SER for Hadlock III, and no significant association was detected for Hadlock II or Shinozuka. The interaction test did not show that its effect differed between the formulas (*p* = 0.274), and its contribution was small (ΔR^2^, 0.006), so the association may be confounded rather than causal and is of little clinical relevance. Sun et al. [10] likewise found no association in 7016 cases scanned within 7 days before delivery. By contrast, Barel et al. [11] reported larger errors and more underestimation in women with diabetes. Diabetes changes fetal body composition as well as weight: these fetuses carry more subcutaneous fat than their weight alone indicates [44], equations that include sonographic abdominal fat outperform Hadlock in diabetic pregnancies [45] and gestational diabetes was associated with larger fetal measurements in a Taiwanese reference study [46].

Because the diabetes variable included two women with type 2 diabetes diagnosed before pregnancy, it is reported as diabetes in pregnancy (242 women with GDM and 2 with pre-existing type 2 diabetes). The distinction matters, because a German series of 1382 deliveries found lower accuracy with pre-existing but not with gestational diabetes [26]. Two women are too few for a separate analysis of pre-existing diabetes, so our findings apply to gestational diabetes and should not be extended to pre-existing diabetes.

*Amniotic fluid volume.* The oligohydramnios group had a lower SER than the normal and polyhydramnios groups with all three formulas, and the interaction test did not detect a difference between the formulas (*p* = 0.497), although with only 29 pregnancies with oligohydramnios, this test had little power. Oligohydramnios tended towards overestimation and polyhydramnios towards underestimation, although the MPE difference was significant only for Shinozuka. This agrees with some reports [11,47]. With little fluid, the fetus lies against the uterine wall, and the abdominal borders are hard to delineate. In our cohort, polyhydramnios was also associated with higher and oligohydramnios with lower birth weight, so part of the effect may again reflect regression towards the mean, although the association persisted after adjustment for birth weight (Table 8). Many other studies found little or no effect of amniotic fluid volume [10,47,48], including a matched study of 57 pregnancies with anhydramnios [49]. Amniotic fluid is also measured imprecisely: automated analysis of 172,293 images detected polyhydramnios with 86.6% sensitivity and 85.7% specificity against expert assessment [50], so some misclassification is likely. Because only 2.3% of pregnancies had oligohydramnios and 3.4% polyhydramnios, the odds ratios were imprecise and the contribution was small (ΔR^2^, 0.009–0.014). The clinical importance is uncertain, but caution is reasonable when the acoustic window is poor.

*Scan-to-delivery interval.* With Hadlock III, scans performed 3 days before delivery had a lower SER than scans at 0–2 days (75.3% versus 84.1–86.3%), but the association was significant only in the Hadlock III model, and the interaction test did not show that it differed between the formulas (*p* = 0.064); the contribution was small (ΔR^2^, 0.017). The literature is inconsistent. Cohen et al. [51] found no difference between scans 0–3 days before delivery, whereas others found scans closer to delivery more accurate [10,52] or, conversely, less accurate [11]. Beyond 7 days, accuracy generally declines [51].

Part of the difference may reflect fetal growth between the scan and delivery rather than error. When the interval cannot be shortened, a gestation-adjusted projection can be applied; a meta-analysis of eight studies reported a MAPE of 8.0% for this approach [53]. In our data, growth played little role (Table 11): scans on the day of delivery were not more accurate (MAPE of Hadlock II, 5.78% versus 5.44% overall), and correcting for growth of 0.5%, 0.8% or 1.0% per day shifted the bias without reducing the error. The lower SER at 3 days rests on only 158 scans and is likely confounded by the reasons for which a woman is rescanned close to delivery, so we do not attribute it to growth.

### 4.4. Strengths and Limitations

To our knowledge, this is the largest study of factors affecting the accuracy of sonographic fetal weight estimation in a Vietnamese population and the first to test the Shinozuka formula in a Southeast Asian cohort. All scans were performed by two sonographers with more than 20 years of experience, gestational age was dated by crown–rump length and the scan-to-delivery interval was at most 3 days. Analysing three formulas in the same pregnancies allowed us to test formally whether a factor acted on all formulas or on only one. By measuring the contribution of each factor, we showed that birth weight had by far the largest association with accuracy, while the other significant factors had very small effects.

The study also has limitations. First, we could not estimate intra- or interobserver reproducibility in this cohort. Biometry was performed in routine care without repeat measurement, and the archived examinations do not permit blinded re-measurement. Operator bias can have large effects. In one quality-review study, a systematic under-measurement of only 0.2 standard deviations led to a 40% over-diagnosis of fetal growth restriction [54], and automated biometry agrees better with experienced than with less experienced operators [55]. Future studies should include repeated, blinded measurements to quantify repeatability and reproducibility, because agreement within and between operators differs between sonographic parameters [56]. Second, the cohort may have been selected. The clinical characteristics of the 1575 excluded pregnancies were not extracted and could not be compared with those of the analysed cohort. The data came from a single private tertiary hospital, and the prevalence of GDM (19.2%) was comparable to that in other Vietnamese hospitals (Section 4.3); accuracy was not lower in these women, but our results apply best to settings with similar screening practices. Gestational and pre-existing diabetes were also recorded as a single variable (Section 4.3). Pregnancies with a non-Vietnamese parent were excluded, so the results should not be extrapolated to the non-Vietnamese population delivering in Vietnamese hospitals. Fetal biometry also differs among Asian populations: Japanese reference values are smaller than the WHO and INTERGROWTH-21st standards and differ from Korean, Chinese, Taiwanese and Thai references [57], and centres in Hong Kong and Taiwan have replaced imported charts with local ones [46,58]. A Japanese formula such as Shinozuka therefore cannot be assumed to suit Vietnamese women. Third, several subgroups remained small after regrouping—39 neonates weighing ≥ 4000 g, 29 pregnancies with oligohydramnios, 47 deliveries before 37 weeks (9 before 34 weeks) and 62 non-cephalic presentations—so the estimates in these groups are imprecise, and the interaction tests had limited power. Fourth, maternal height and weight were analysed in categories, pre-pregnancy body mass index was not available and birth weight, the strongest factor in our models, is unknown before delivery; our models therefore describe associations and were not designed to improve or replace the formulas. Finally, we did not record other potentially influential factors, such as pre-pregnancy body mass index, placental location, machine and image quality, measurement technique and sonographer experience.

## 5. Conclusions

Fetal size was the factor most strongly associated with the accuracy of sonographic fetal weight estimation with the Hadlock II, Hadlock III and Shinozuka formulas: against birth weight, all three overestimated the weight of small fetuses and underestimated that of large ones. Part of this pattern reflects regression towards the mean, and because only the EFW is known before birth, it cannot be used as a bedside correction. Amniotic fluid volume, diabetes in pregnancy, the scan-to-delivery interval and gestational age were also associated with accuracy in some models, but removing any one of them reduced the Nagelkerke R^2^ by less than 0.02; no independent association with the SER was found for maternal height, maternal weight at delivery, fetal sex or presentation in the models used. In these models, maternal characteristics showed little additional association with accuracy after accounting for the other variables; whether they could improve the formulas would require a separate analysis with validation. The differences between the three formulas were small, and their clinical relevance is unclear; none can be recommended over the others. For clinicians, the take-home message is this: an EFW below 2500 g or of ≥4000 g is usually correct, but an EFW of 2500–2999 g does not exclude a low-birth-weight neonate, and an EFW of 3500–3999 g does not exclude a neonate of ≥4000 g, particularly when there are risk factors for macrosomia. The same caution is needed in the presence of oligohydramnios.

## Figures and Tables

**Table 1:** Fetal weight estimation formulas applied in the study.

Formula	Year	Study Population	Parameters	Equation
Hadlock II [4]	1985	USA	BPD, AC, FL	log10(EFW) = 1.335 + 0.0316·BPD + 0.0457·AC + 0.1623·FL − 0.0034·AC·FL
Hadlock III [4]	1985	USA	HC, AC, FL	log10(EFW) = 1.326 + 0.0438·AC + 0.158·FL + 0.0107·HC − 0.00326·AC·FL
Shinozuka [5]	2000	Japan	BPD, AC, FL	EFW = 1.07·BPD^3^ + 0.30·AC^2^·FL

AC, abdominal circumference; BPD, biparietal diameter; EFW, estimated fetal weight; FL, femur length; HC, head circumference. Measurements are in centimetres.

**Table 2:** Demographic, pregnancy and ultrasound characteristics of the study population (*n* = 1262).

Characteristic	Value
Maternal age (years)	32.7 ± 4.2 (17–49)
Maternal height (cm)	158.8 ± 5.2 (143–175)
<150	19 (1.5)
150–159.9	666 (52.8)
160–169.9	540 (42.8)
≥170	37 (2.9)
Maternal weight at delivery (kg)	67.0 ± 8.6 (48.3–115.4)
<50	9 (0.7)
50–59.9	244 (19.3)
60–69.9	608 (48.2)
70–79.9	307 (24.3)
80–89.9	75 (5.9)
≥90	19 (1.5)
Diabetes in pregnancy ^a^	
No	1018 (80.7)
Yes	244 (19.3)
Gestational age (weeks)	38.8 ± 1.04 (30–41)
30–33 + 6	9 (0.7)
34–36 + 6	38 (3.0)
37–39 + 6	1114 (88.3)
40–41	101 (8.0)
Fetal sex	
Male	645 (51.1)
Female	617 (48.9)
Birth weight (g)	3150 ± 429 (1570–4752)
<2500	89 (7.1)
2500–2999	360 (28.5)
3000–3499	573 (45.4)
3500–3999	201 (15.9)
≥4000	39 (3.1)
Scan-to-delivery interval (days)	
0	246 (19.5)
1	581 (46.0)
2	277 (21.9)
3	158 (12.5)
Amniotic fluid	
Normal	1190 (94.3)
Polyhydramnios	43 (3.4)
Oligohydramnios	29 (2.3)
Fetal presentation	
Cephalic	1200 (95.1)
Non-cephalic	62 (4.9)

Values are presented as mean ± SD (range) or *n* (%). ^a^ 242 women with gestational diabetes mellitus and 2 with type 2 diabetes diagnosed before pregnancy. Percentages may not sum to 100% because of rounding.

**Table 3:** Overall accuracy of the Hadlock II, Hadlock III and Shinozuka formulas in the whole cohort (*n* = 1262).

Formula	MPE (%), Mean ± SD	MAPE (%), Mean ± SD	SER (APE ≤ 10%), *n* (%)	APE ≤ 5%, *n* (%)	APE ≤ 15%, *n* (%)
Hadlock II	−0.37 ± 7.05	5.44 ± 4.49	1088 (86.2)	687 (54.4)	1214 (96.2)
Hadlock III	−1.47 ± 7.05	5.64 ± 4.48	1062 (84.2)	657 (52.1)	1210 (95.9)
Shinozuka	+1.83 ± 7.26	5.75 ± 4.79	1067 (84.5)	668 (52.9)	1201 (95.2)

APE, absolute percentage error; MAPE, mean absolute percentage error; MPE, mean percentage error; SD, standard deviation; SER, successful estimation rate.

**Table 4:** Paired comparison of the three formulas in the same 1262 pregnancies.

Comparison	Δ MAPE, pp (95% CI)	Wilcoxon *p* *	Δ SER, pp (95% CI)	McNemar *p* *
Hadlock II vs. Hadlock III	−0.20 (−0.30 to −0.10)	<0.001	+2.06 (+0.71 to +3.41)	0.012
Hadlock II vs. Shinozuka	−0.31 (−0.45 to −0.18)	<0.001	+1.66 (+0.11 to +3.22)	0.093
Hadlock III vs. Shinozuka	−0.11 (−0.30 to +0.08)	0.431	−0.40 (−2.40 to +1.61)	0.757

CI, confidence interval; MAPE, mean absolute percentage error; pp, percentage points; SER, successful estimation rate (absolute percentage error ≤ 10%). Δ is the value for the first formula minus that for the second. Confidence intervals for Δ MAPE were obtained from 10,000 bootstrap resamples; those for Δ SER are paired Wald intervals. * Holm-adjusted for three comparisons; confidence intervals are not adjusted. Across the three formulas, Friedman χ^2^ = 17.48, df = 2, *p* < 0.001 for the absolute percentage error and Cochran’s Q = 6.64, df = 2, *p* = 0.036 for the successful estimation rate.

**Table 5:** Bias, 95% limits of agreement and Deming regression of the estimated on the actual fetal weight (*n* = 1262).

**(A) Bias, fixed limits of agreement and Deming regression**				
**Formula**	**Bias, g (95% CI)**	**Lower 95% LoA (95% CI)**	**Upper 95% LoA (95% CI)**	**Deming Slope (95% CI)**
Hadlock II	−25.6 (−37.7 to −13.5)	−455.1 (−476.1 to −434.2)	+403.9 (+383.0 to +424.9)	0.853 (0.821–0.882)
Hadlock III	−61.5 (−73.8 to −49.2)	−498.8 (−520.1 to −477.4)	+375.8 (+354.4 to +397.1)	0.817 (0.788–0.848)
Shinozuka	+39.4 (+27.2 to +51.7)	−394.5 (−415.6 to −373.3)	+473.3 (+452.2 to +494.5)	0.792 (0.761–0.823)
(**B**) **Regression-based limits, g (% of BW)**				
**Formula**	**At 2500 g**	**At 3150 g (mean)**	**At 4000 g**	**Slope of absolute residuals (95% CI)**
Hadlock II	+137 (−233 to +508); −9.3% to +20.3%	−26 (−392 to +341); −12.5% to +10.8%	−238 (−600 to +124); −15.0% to +3.1%	−0.0023 (−0.0176 to +0.0130)
Hadlock III	+121 (−241 to +483); −9.6% to +19.3%	−62 (−424 to +301); −13.5% to +9.6%	−300 (−663 to +62); −16.6% to +1.6%	+0.0001 (−0.0148 to +0.0151)
Shinozuka	+233 (−126 to +593); −5.0% to +23.7%	+39 (−307 to +386); −9.7% to +12.2%	−214 (−543 to +115); −13.6% to +2.9%	−0.0081 (−0.0226 to +0.0064)
(**C**) **Paired difference in slope**				
**Comparison**	**Deming slope (95% CI)**		**Bland–Altman slope (95% CI)**	
Hadlock II − Hadlock III	0.035 (0.027 to 0.043)		0.031 (0.024 to 0.037)	
Hadlock II − Shinozuka	0.061 (0.056 to 0.066)		0.048 (0.044 to 0.052)	
Hadlock III − Shinozuka	0.026 (0.016 to 0.036)		0.017 (0.009 to 0.026)	

CI, confidence interval; LoA, limits of agreement. (**A**) Bias is the mean of (EFW − BW). The confidence interval for each limit of agreement was calculated as the limit ± t_0.975;_*_n_*_−1_ × SD × √(3/*n*). Deming regression was fitted with an error-variance ratio λ = 1; confidence intervals were obtained from 2000 bootstrap resamples. The Deming intercepts at 0 g (+439, +514 and +696 g) are extrapolations and were not interpreted; at the mean birth weight the Deming line equals the bias. (**B**) Expected difference (95% limits) in grams, then the limits as a percentage of birth weight. The slope of the absolute residuals is in g per g of birth weight. (**C**) Differences between the slopes of two formulas, estimated in the same 2000 bootstrap resamples of women; a positive value means that the second formula has the slope further from unity (Deming) or the steeper negative slope (Bland–Altman). BW, birth weight; EFW, estimated fetal weight.

**Table 6:** Accuracy by each formula’s own estimated fetal weight group and identification of low-birth-weight neonates and neonates weighing ≥ 4000 g (*n* = 1262).

(**A**) **Accuracy by estimated-weight group**			
**Estimated-Weight Group (g)**	**Hadlock II** ***n*** **/MPE/SER**	**Hadlock III** ***n*** **/MPE/SER**	**Shinozuka** ***n*** **/MPE/SER**
<2500	74/−2.12/79.7	76/−2.26/77.6	45/+0.44/84.4
2500–2999	364/−0.50/83.5	407/−1.63/83.0	283/+2.61/77.7
3000–3499	637/−0.05/87.3	634/−1.16/85.2	714/+2.09/86.1
3500–3999	171/−0.28/89.5	137/−1.91/85.4	208/+0.38/87.5
≥4000	16/−2.61/100.0	8/−2.92/100.0	12/−2.21/100.0
Slope of the difference on estimated weight (95% CI)	+0.013 (−0.019 to +0.046); *p* = 0.418	−0.016 (−0.050 to +0.019); *p* = 0.372	−0.049 (−0.083 to −0.014); *p* = 0.006
Slope of the difference on birth weight	−0.250; *p* < 0.001	−0.281; *p* < 0.001	−0.298; *p* < 0.001
(**B**) **Identification by weight threshold**			
**Criterion**	**Hadlock II**	**Hadlock III**	**Shinozuka**
Neonates < 2500 g with EFW < 2500 g (sensitivity)	58/89 (65.2; 54.3–75.0)	59/89 (66.3; 55.5–76.0)	37/89 (41.6; 31.2–52.5)
EFW < 2500 g confirmed as neonates < 2500 g (PPV)	58/74 (78.4; 67.3–87.1)	59/76 (77.6; 66.6–86.4)	37/45 (82.2; 67.9–92.0)
Neonates ≥ 4000 g with EFW ≥ 4000 g (sensitivity)	15/39 (38.5; 23.4–55.4)	7/39 (17.9; 7.5–33.5)	12/39 (30.8; 17.0–47.6)
EFW ≥ 4000 g confirmed as neonates ≥ 4000 g (PPV)	15/16 (93.8; 69.8–99.8)	7/8 (87.5; 47.3–99.7)	12/12 (100.0; 73.5–100.0)
Neonates ≥ 4000 g with EFW 3500–3999 g	23/39 (59.0)	30/39 (76.9)	26/39 (66.7)

MPE, mean percentage error (%); SER, successful estimation rate (%; absolute percentage error ≤ 10%); difference, EFW − BW (g); PPV, positive predictive value; CI, confidence interval. In panel (**A**), each formula is grouped by its own estimated weight. Panel (**B**) shows *n*/N (%; exact 95% CI). The ≥4000 g estimated-weight group was very small (8–16 cases), so the figures at this level are descriptive only.

**Table 7:** Univariate analysis of factors affecting the accuracy of the fetal weight estimation formulas.

Factor	Group	Hadlock II			Hadlock III			Shinozuka		
		MPE	MAPE	SER	MPE	MAPE	SER	MPE	MAPE	SER
Maternal height (cm)	<150	0.05 ± 8.63 *	6.05 ± 5.98	84.2	−0.79 ± 8.65 *	6.48 ± 5.59 *	78.9	2.16 ± 8.69 *	6.72 ± 5.73	78.9
	150–159.9	0.01 ± 6.92 *	5.25 ± 4.50	85.7	−0.95 ± 6.88 *	5.34 ± 4.45 *	84.4	2.22 ± 7.18 *	5.73 ± 4.86	85.4
	160–169.9	−0.57 ± 7.12 *	5.62 ± 4.40	87.6	−1.82 ± 7.13 *	5.88 ± 4.41 *	84.6	1.60 ± 7.30 *	5.78 ± 4.74	83.5
	≥170	−4.50 ± 6.08 *	5.89 ± 4.71	75.7	−6.19 ± 6.23 *	7.14 ± 5.08 *	75.7	−2.12 ± 6.26 *	5.24 ± 3.95	86.5
Maternal weight (kg)	<50	2.34 ± 7.58 *	6.46 ± 4.07	88.9	2.37 ± 8.30 *	6.95 ± 4.56	77.8	4.99 ± 7.42 *	7.85 ± 3.69	66.7
	50–59.9	0.27 ± 7.40 *	5.49 ± 4.96	84.0	−0.56 ± 7.30 *	5.49 ± 4.83	84.4	2.84 ± 7.51 *	5.87 ± 5.47	84.0
	60–69.9	−0.45 ± 6.86 *	5.38 ± 4.27	86.7	−1.63 ± 6.90 *	5.62 ± 4.32	84.5	1.83 ± 7.10 *	5.74 ± 4.54	84.2
	70–79.9	−0.28 ± 7.16 *	5.48 ± 4.61	86.6	−1.40 ± 7.11 *	5.67 ± 4.49	84.7	1.68 ± 7.27 *	5.70 ± 4.81	85.0
	80–89.9	−2.26 ± 6.95 *	5.72 ± 4.51	85.3	−3.69 ± 7.05 *	6.25 ± 4.88	77.3	−0.84 ± 7.36 *	5.70 ± 4.69	88.0
	≥90	−1.22 ± 5.39 *	4.36 ± 3.24	94.7	−2.34 ± 5.25 *	4.72 ± 3.13	89.5	0.47 ± 6.11 *	4.65 ± 3.84	89.5
Diabetes in pregnancy	No	−0.24 ± 7.19	5.53 ± 4.60	85.5	−1.34 ± 7.18	5.73 ± 4.53	83.1 *	2.01 ± 7.38	5.86 ± 4.91	84.3
	Yes	−0.92 ± 6.41	5.08 ± 4.02	89.3	−2.00 ± 6.47	5.27 ± 4.24	88.5 *	1.06 ± 6.73	5.30 ± 4.26	85.7
Birth weight (g)	<2500	4.75 ± 9.33 *	7.58 ± 7.19 *	70.8 *	4.12 ± 8.97 *	7.44 ± 6.46 *	74.2*	9.19 ± 8.99 *	10.12 ± 7.93 *	60.7 *
	2500–2999	3.02 ± 6.89 *	5.85 ± 4.73 *	83.6 *	2.16 ± 6.69 *	5.40 ± 4.50 *	84.4 *	5.88 ± 6.61 *	7.29 ± 5.01 *	75.8 *
	3000–3499	−1.27 ± 5.61 *	4.57 ± 3.49 *	92.0 *	−2.43 ± 5.51 *	4.83 ± 3.60 *	90.6 *	0.69 ± 5.39 *	4.26 ± 3.36 *	92.7 *
	3500–3999	−4.71 ± 5.49 *	5.75 ± 4.38 *	84.6 *	−6.20 ± 5.59 *	6.84 ± 4.78 *	75.1 *	−3.61 ± 5.09 *	4.90 ± 3.86 *	90.0 *
	≥4000	−7.63 ± 5.37 *	7.98 ± 4.83 *	69.2 *	−9.30 ± 5.12 *	9.50 ± 4.72 *	56.4 *	−7.63 ± 5.10 *	7.89 ± 4.68 *	71.8 *
Gestational age (weeks)	<34	−7.26 ± 6.59 *	7.48 ± 6.31	66.7	−7.73 ± 6.09 *	8.48 ± 4.84 *	66.7	−2.09 ± 7.10	5.53 ± 4.58	88.9
	34–36 + 6	−3.41 ± 7.99 *	6.72 ± 5.43	81.6	−3.69 ± 8.30 *	7.45 ± 5.08 *	73.7	0.41 ± 8.16	6.25 ± 5.16	81.6
	37–39 + 6	−0.34 ± 6.98 *	5.40 ± 4.43	86.4	−1.49 ± 7.00 *	5.60 ± 4.45 *	84.4	1.80 ± 7.23	5.74 ± 4.74	85.0
	40–41	1.13 ± 6.86 *	5.22 ± 4.56	88.1	0.18 ± 6.79 *	5.13 ± 4.41 *	87.1	3.02 ± 7.19	5.76 ± 5.24	80.2
Fetal sex	Male	−0.99 ± 7.16 *	5.58 ± 4.60	86.4	−2.14 ± 7.18 *	5.87 ± 4.65	82.8	1.24 ± 7.45 *	5.83 ± 4.80	85.6
	Female	0.28 ± 6.87 *	5.29 ± 4.38	86.1	−0.77 ± 6.86 *	5.40 ± 4.29	85.6	2.44 ± 7.02 *	5.68 ± 4.79	83.5
Presentation	Cephalic	−0.23 ± 6.99 *	5.39 ± 4.45	86.6	−1.40 ± 7.03	5.61 ± 4.46	84.3	1.95 ± 7.20 *	5.73 ± 4.78	84.6
	Non-cephalic	−3.10 ± 7.64 *	6.37 ± 5.18	79.0	−2.92 ± 7.36	6.23 ± 4.83	80.6	−0.53 ± 7.99 *	6.22 ± 4.99	83.9
Amniotic fluid	Normal	−0.39 ± 6.88	5.35 ± 4.34	86.9 *	−1.50 ± 6.89	5.54 ± 4.36 *	85.0 *	1.83 ± 7.06 *	5.66 ± 4.61	85.2 *
	Polyhydramnios	−1.28 ± 9.19	6.35 ± 6.70	86.0 *	−2.39 ± 9.18	6.88 ± 6.45 *	79.1 *	−0.19 ± 9.78 *	6.56 ± 7.19	81.4 *
	Oligohydramnios	1.81 ± 9.60	7.55 ± 6.05	58.6 *	1.09 ± 9.39	7.77 ± 5.18 *	58.6 *	4.76 ± 9.83 *	8.47 ± 6.77	62.1 *
Scan-to-delivery interval (days)	0	−0.08 ± 7.43 *	5.78 ± 4.65 *	85.0	−1.20 ± 7.33 *	5.85 ± 4.57 *	84.1 *	2.27 ± 7.75 *	6.27 ± 5.07	80.5
	1	−0.12 ± 7.12 *	5.29 ± 4.76 *	86.1	−1.17 ± 7.10 *	5.47 ± 4.67 *	85.5 *	1.98 ± 7.27 *	5.60 ± 5.04	85.7
	2	−0.09 ± 6.35 *	5.12 ± 3.75 *	88.8	−1.09 ± 6.44 *	5.27 ± 3.84 *	86.3 *	2.08 ± 6.52 *	5.46 ± 4.10	87.4
	3	−2.24 ± 7.09 *	6.00 ± 4.36 *	84.2	−3.66 ± 7.14 *	6.58 ± 4.57 *	75.3 *	0.14 ± 7.52 *	6.01 ± 4.49	81.6

MAPE, mean absolute percentage error (%); MPE, mean percentage error (%); SER, successful estimation rate (%; absolute percentage error ≤ 10%). MPE and MAPE are presented as mean ± SD and expressed as percentages. The Mann–Whitney U test (two groups) or the Kruskal–Wallis test (more than two groups) was used for MPE and MAPE; the chi-square or Fisher exact test was used for SER. * *p* < 0.05.

**Table 8:** Multivariable logistic regression of factors associated with the successful estimation rate of each formula.

(**A**) **Hadlock II**					
		**OR**	**95% CI**	***p***	**ΔR^2^ (×100)**
Birth weight (g)	<2500	1.33	0.57–3.10	0.505	5.2
	2500–2999	2.37	1.12–4.99	0.024	
	3000–3499	5.40	2.54–11.47	<0.001	
	3500–3999	2.51	1.14–5.49	0.022	
	≥4000	Reference			
Amniotic fluid	Normal	3.94	1.76–8.84	<0.001	1.2
	Polyhydramnios	4.38	1.31–14.59	0.016	
	Oligohydramnios	Reference			
Model Nagelkerke R^2^ (×100)					7.4
(**B**) **Hadlock III**					
		**OR**	**95% CI**	***p***	**ΔR^2^ (×100)**
Birth weight (g)	<2500	2.86	1.25–6.55	0.013	6.4
	2500–2999	4.56	2.23–9.31	<0.001	
	3000–3499	8.17	4.01–16.61	<0.001	
	3500–3999	2.38	1.16–4.90	0.018	
	≥4000	Reference			
Amniotic fluid	Normal	4.22	1.88–9.43	<0.001	1.4
	Polyhydramnios	4.22	1.36–13.11	0.013	
	Oligohydramnios	Reference			
Diabetes in pregnancy	No	0.62	0.40–0.97	0.036	0.6
	Yes	Reference			
Scan-to-delivery interval (days)	0	2.06	1.22–3.47	0.007	1.7
	1	2.22	1.42–3.47	<0.001	
	2	2.29	1.37–3.85	0.002	
	3	Reference			
Model Nagelkerke R^2^ (×100)					10.7
(**C**) **Shinozuka**					
		**OR**	**95% CI**	***p***	**ΔR^2^ (×100)**
Birth weight (g)	<2500	0.49	0.20–1.16	0.104	12.9
	2500–2999	1.12	0.52–2.40	0.769	
	3000–3499	5.11	2.34–11.20	<0.001	
	3500–3999	3.66	1.56–8.55	0.003	
	≥4000	Reference			
Gestational age (weeks)	<37	5.70	2.07–15.70	<0.001	1.5
	37–39 + 6	2.15	1.23–3.73	0.007	
	40–41	Reference			
Amniotic fluid	Normal	2.79	1.16–6.71	0.022	0.9
	Polyhydramnios	1.70	0.51–5.65	0.383	
	Oligohydramnios	Reference			
Scan-to-delivery interval (days)	0	1.02	0.59–1.76	0.947	0.8
	1	1.42	0.87–2.32	0.164	
	2	1.77	1.00–3.13	0.049	
	3	Reference			
Model Nagelkerke R^2^ (×100)					15.3

CI, confidence interval; OR, odds ratio; ΔR^2^, reduction in Nagelkerke R^2^ (×100) when the factor is removed from the full nine-factor enter model; Nagelkerke R^2^ is a pseudo-R^2^ and not a proportion of explained variance. The reference category for each factor is indicated. Only factors retained in each final backward-stepwise model are shown. Gestational age was entered as a single category below 37 weeks, and maternal height and weight in the combined strata defined in Section 2.3, to avoid unstable estimates in sparse cells. (**A**) Hadlock II, (**B**) Hadlock III and (**C**) Shinozuka: final model for each formula.

**Table 9:** Generalised estimating equations (GEE) model with formula as a within-woman repeated factor (3786 estimates in 1262 women).

(**A**) **Effect of Formula**			
	**Adjusted OR**	**95% CI**	***p***
Hadlock II	Reference		
Hadlock III	0.84	0.75–0.94	0.003
Shinozuka	0.87	0.76–0.99	0.037
(**B**) **Tests of factor × formula interaction**			
	**Wald χ^2^ (df)**	***p***	***p*** **(Holm)**
Birth weight	68.6 (8)	<0.001	<0.001
Gestational age	10.0 (4)	0.040	0.266
Maternal height	12.5 (4)	0.014	0.114
Maternal weight at delivery	7.2 (6)	0.307	0.821
Diabetes in pregnancy	2.6 (2)	0.274	0.821
Fetal sex	6.5 (2)	0.038	0.266
Presentation	4.6 (2)	0.098	0.393
Amniotic fluid	3.4 (4)	0.497	0.821
Scan-to-delivery interval	11.9 (6)	0.064	0.318
(**C**) **OR for each birth-weight group, by formula**			
	**Hadlock II**	**Hadlock III**	**Shinozuka**
<2500 g	0.22 (0.12–0.40)	0.31 (0.17–0.58)	0.12 (0.07–0.22)
2500–2999 g	0.43 (0.28–0.66)	0.55 (0.36–0.83)	0.24 (0.16–0.36)
3000–3499 g	Reference	Reference	Reference
3500–3999 g	0.45 (0.27–0.75)	0.29 (0.19–0.45)	0.67 (0.38–1.19)
≥4000 g	0.18 (0.08–0.39)	0.12 (0.06–0.25)	0.18 (0.08–0.41)

The outcome is a successful estimate (absolute percentage error ≤ 10%). GEE model with binomial family, logit link, exchangeable correlation (ρ = 0.61), woman as the clustering unit and robust standard errors; all models are adjusted for the nine factors. (**A**) Adjusted odds ratio of a successful estimate with each formula, relative to Hadlock II. (**B**) Each interaction term was added one factor at a time; df = (number of categories of the factor − 1) × 2. All factors were coded in the merged strata of Table 8 (gestational age < 37, 37–39 + 6 and ≥40 weeks; maternal height < 160, 160–169.9 and ≥170 cm; maternal weight < 60, 60–69.9, 70–79.9 and ≥80 kg). Sensitivity analysis: with gestational age in four categories (<34, 34–36 + 6, 37–39 + 6 and ≥40 weeks), the gestational age × formula interaction was significant (χ^2^ = 19.4, df = 6, *p* = 0.004; Holm-adjusted *p* = 0.028), driven by nine pregnancies before 34 weeks; the results for the other factors were unchanged. (**C**) Estimates from the model with the birth weight × formula interaction, with 3000–3499 g as the reference; OR (95% CI). CI, confidence interval; OR, odds ratio.

**Table 10:** Contribution of each factor (ΔR^2^ × 100) under three definitions of the outcome, for the same nine-factor model, with the merged strata used in Table 8 (*n* = 1262).

Formula and Outcome	Full Model R^2^ (×100)	Four Largest Contributors (ΔR^2^ × 100)
Hadlock II		
SER ≤ 10% (logistic, Nagelkerke R^2^)	8.8	birth weight 5.2, amniotic fluid 1.2, maternal height 0.4, interval 0.4
Absolute percentage error (linear R^2^)	5.9	birth weight 3.8, interval 0.5, amniotic fluid 0.3, maternal height 0.2
Absolute error in grams (linear R^2^)	8.3	birth weight 6.2, interval 0.6, amniotic fluid 0.2, maternal height 0.2
Hadlock III		
SER ≤ 10% (logistic, Nagelkerke R^2^)	11.3	birth weight 6.4, interval 1.7, amniotic fluid 1.4, diabetes in pregnancy 0.6
Absolute percentage error (linear R^2^)	8.5	birth weight 5.0, interval 0.9, maternal height 0.5, amniotic fluid 0.4
Absolute error in grams (linear R^2^)	15.6	birth weight 11.8, interval 1.1, maternal height 0.6, amniotic fluid 0.3
Shinozuka		
SER ≤ 10% (logistic, Nagelkerke R^2^)	16.0	birth weight 12.9, gestational age 1.5, amniotic fluid 0.9, interval 0.8
Absolute percentage error (linear R^2^)	16.6	birth weight 15.0, gestational age 1.3, amniotic fluid 0.4, interval 0.4
Absolute error in grams (linear R^2^)	11.5	birth weight 10.1, gestational age 1.1, interval 0.4, amniotic fluid 0.3

ΔR^2^ is the reduction in the model R^2^ when the factor is removed from the full nine-factor enter model. The absolute error in grams does not contain birth weight in a denominator and is therefore free of the ratio-scale coupling that affects the percentage error. Interval, scan-to-delivery interval; SER, successful estimation rate. Nagelkerke R^2^ and linear R^2^ are not directly comparable; only the ranking of factors within the same outcome definition should be compared.

**Table 11:** Accuracy of the three formulas by scan-to-delivery interval and after correction for expected fetal growth.

Analysis	*n*	Hadlock II (MPE/MAPE/SER)	Hadlock III (MPE/MAPE/SER)	Shinozuka (MPE/MAPE/SER)
By scan-to-delivery interval				
0–3 days (main analysis)	1262	−0.37/5.44/86.2	−1.47/5.64/84.2	+1.83/5.75/84.5
≤2 days	1104	−0.10/5.36/86.5	−1.16/5.51/85.4	+2.07/5.72/85.0
≤1 day	827	−0.10/5.44/85.7	−1.18/5.58/85.1	+2.07/5.80/84.2
Same day as delivery	246	−0.08/5.78/85.0	−1.20/5.85/84.1	+2.27/6.27/80.5
After growth correction				
EFW × (1 + 0.005 × days)	1262	+0.26/5.44/86.7	−0.85/5.56/85.5	+2.48/5.93/83.6
EFW × (1 + 0.008 × days) ^a^	1262	+0.64/5.47/86.5	−0.47/5.54/86.1	+2.86/6.07/82.3
EFW × (1 + 0.010 × days)	1262	+0.90/5.51/86.1	−0.22/5.54/86.1	+3.12/6.16/81.4

MPE, mean percentage error (%); MAPE, mean absolute percentage error (%); SER, successful estimation rate (%; absolute percentage error ≤ 10%). ^a^ The main factor of 0.8% per day is based on the near-linear third-trimester fetal growth reported in 12,425 term singleton pregnancies [20]. The factors 0.005 and 0.010 were included to test the robustness of the conclusions to uncertainty in the true growth rate.

## Data Availability

The de-identified analysis dataset is available on reasonable request from the corresponding author; it is not publicly available because it contains information that could compromise the privacy of the participants.

## Abbreviations

The following abbreviations are used in this manuscript:

AC	abdominal circumference
AFI	amniotic fluid index
APE	absolute percentage error
aRR	adjusted risk ratio
BPD	biparietal diameter
BW	birth weight
CI	confidence interval
EFW	estimated fetal weight
FL	femur length
GDM	gestational diabetes mellitus
GEE	generalised estimating equations
HC	head circumference
ISUOG	International Society of Ultrasound in Obstetrics and Gynecology
LoA	limits of agreement
MAPE	mean absolute percentage error
MPE	mean percentage error
OR	odds ratio
PE	percentage error
PPV	positive predictive value
SD	standard deviation
SDP	single deepest pocket
SER	successful estimation rate
STROBE	Strengthening the Reporting of Observational Studies in Epidemiology
